# Evaluation of the Entomological Adaptive Surveillance Framework for malaria vector monitoring: a comparative field trial with routine surveillance in Ghana and Mozambique

**DOI:** 10.1136/bmjph-2025-004060

**Published:** 2026-03-31

**Authors:** Luigi Sedda, Abdollah Jalilian, Mercy Opiyo, Steven Gowelo, Dulcisária J Marrenjo, Christian Atta-Obeng, Ernest Boampong, Samuel K Oppong, Otubea Owusu-Akrofi, Edward Thomsen, Allison Tatarsky, Keziah L Malm, Baltazar Candrinho, Neil F Lobo

**Affiliations:** 1 Lancaster Ecology and Epidemiology Group, Lancaster Medical School, Lancaster University, Lancaster, UK; 2 Malaria Elimination Initiative, Institute for Global Health Sciences, University of California San Francisco, San Francisco, California, USA; 3 Centro de Investigação em Saúde de Manhiça, Manhica, Maputo Province, Mozambique; 4 Programa Nacional de Controlo da Malária, Ministerio da Saude, Maputo, Maputo City, Mozambique; 5 National Malaria Elimination Programme, Ghana Ministry of Health, Accra, Greater Accra Region, Ghana; 6 University of Notre Dame, Notre Dame, Indiana, USA

**Keywords:** Statistics as Topic, Sentinel Surveillance, Disease Vectors

## Abstract

**Introduction:**

Accurate mosquito surveillance is essential for guiding targeted interventions. To capture the spatial and temporal heterogeneity of mosquito populations, surveillance designs would ideally be flexible and evidence-based, that is, informed by prior data. This study compares a newly developed entomological adaptive surveillance framework (EASF) and routine entomological surveillance in Ghana and Mozambique. Routine surveillance refers to standard practices by National Malaria Control Programmes, while EASF enables the strategic selection of sampling locations based on modelled risk or environmental criteria. The study evaluates both approaches based on *Anopheles* mosquito catch rates, model predictive performance and the proportion of obsolete sampling locations—those contributing little to overall surveillance outcomes and model improvement.

**Methods:**

A Bayesian framework for exceedance probability is employed in this adaptive design to select the number and location of the adaptive sites. Estimates are based on a Bayesian spatiotemporal model with nugget effects to analyse mosquito abundance, using log-transformed counts to address heavy-tailed distributions.

**Results:**

The EASF outperformed routine surveillance in most metrics, including mosquito catch rates, model robustness and reduction in uncertainty. Notably, when standardised, EASF sites yielded higher mosquito catches and more stable predictions, as indicated by lower coefficients of variation. While EASF generally improved model inference and predictive accuracy, performance varied by country. Nevertheless, EASF consistently identified proportionally fewer obsolete locations than routine surveillance, demonstrating its efficiency in targeting informative sites.

**Conclusions:**

EASF offers an effective, evidence-based approach to improving surveillance precision, enabling surveillance programmes to dynamic transmission systems and emerging needs while maintaining operational feasibility. Integrating adaptive and routine designs can enhance surveillance efficiency, either by improving accuracy, reducing site numbers or accelerating detection of ecological changes. The key to effective entomological surveillance is not rigidly achieving a target, but continuously adapting towards it.

**WHAT IS ALREADY KNOWN ON THIS TOPIC**

- Mosquito surveillance in malaria-endemic countries has improved significantly, yet current systems remain insufficient to meet emerging challenges, despite WHO promoting it as a core intervention to guide targeted control strategies and monitor disease trends.
- To overcome current limitations, it is essential to develop surveillance designs that yield accurate estimates of mosquito distribution while remaining feasible within constrained resources.

**WHAT THIS STUDY ADDS**

- This study demonstrates that adaptive surveillance designs can significantly improve the accuracy and efficiency of mosquito monitoring compared with routine approaches, even when operating under resource constraints.

**HOW THIS STUDY MIGHT AFFECT RESEARCH, PRACTICE OR POLICY**

- By adopting or integrating adaptive surveillance frameworks, National Malaria Control Programmes can implement more cost-effective strategies that enhance data quality and support timely, evidence-based vector control decisions.

## Introduction

 Since 2000, global malaria control efforts have averted an estimated 2.2 billion cases and 12.7 million deaths, 95% of which have been in Africa.^1^ However, there remain significant challenges, as highlighted by the increase of 11 million cases in 2023 compared with 2022. There is an urgent need for equitable access to life-saving tools, such as next-generation bed nets, improved treatment options and the WHO-recommended malaria vaccine, which has already been introduced in 17 countries.^2^ Funding gaps require subnational prioritisation of resources and interventions to reduce cases, and at the same time, prevent the re-establishment of malaria in elimination zones. The WHO identifies vector control and entomological surveillance as key strategies to minimise the risk of malaria transmission and as tools for intervention prioritisation.

Accurate assessments of mosquito occurrence and dynamics are essential for the efficient deployment of disease surveillance and control interventions. To obtain unbiased or representative information on mosquito species, behaviour and ecology—including how these relate to environmental factors over time and space—sampling must cover the entire geographical area, including locations where mosquito-environment relationships may be weak.^3^

Due to the high spatial and temporal heterogeneity of mosquito-borne diseases, the surveillance design needs to be flexible to represent the dynamicity of the disease system components.^4^ When previous data are available, sampling may be based on a target function through model or risk-based approaches (with risk evolving in space and time^5^). This allows the collection of adequate, representative and targeted data with limited resources.^6^ Such approaches are a form of preferential sampling and are variously called ‘unequal probability survey designs’ or ‘adaptive designs’. They allow the selection of locations based on specific criteria (eg, to maximise the accuracy of the mosquito distribution, or to determine the effects of environmental factors on mosquito bionomics), and locations are selected based on different probabilities of appearing in the sample, for example, if the locations are more likely to be selected from areas with a certain target function value.^7^ In an adaptive spatial sampling scheme, at each step, the data analysis is executed using an enriched sample data set^8^ resulting from the inclusion of new locations, and in some cases, the removal of locations whose information has reached saturation. For example, target functions or measures can be associated with the predictive accuracy (eg, root mean square error) or probability of detection (eg, areas with probability of mosquito presence above 0.2).^9^ Improving detection of mosquito (increasing the likelihood of finding them) or disease ecological corridors could allow targeting of interventions to these areas to obtain the maximum gains in disease control and elimination.

A common choice for adaptive sampling or surveillance (hereafter when ‘sampling’ refers to a design, it is replaced by the word ‘surveillance’) is a Bayesian framework for exceedance probability. The advantages of this approach are (1) it allows estimates of uncertainty in the model inference and in prediction of the outcome variable; and (2) the flexibility to include a spatial or spatiotemporal structure in different model components. This approach is also used in spatial disease cluster detection, and it is considered a superior method in comparison with other clustering techniques.^10^

With dwindling resources available to support malaria control and elimination efforts, it is becoming increasingly important to maximise the efficiency of surveillance strategies, especially when surveillance may be considered an intervention. Tools to guide the development of locally-tailored and question-based vector surveillance approaches are available,^11 12^ but these frameworks do not include evidence-based guidance on adaptive surveillance. This study aimed to compare adaptive and routine surveillance designs in Ghana and Mozambique. In this context, ‘routine surveillance’ refers to the standard entomological surveillance activities being conducted by the National Malaria Control Programme. The study had the following objectives:

1. To compare the data and models from the Routine and Adaptive designs in terms of: (1) average number of mosquitoes collected (considering sampling effort); (2) predictive ability, or the capacity of the model to provide accurate estimations outside the study area; and (3) the quality of the information provided by the surveillance locations as measured by the proportion of obsolete locations—locations which data does not improve model inference and estimation.
2. To quantify the gains of employing an adaptive approach in terms of improved model inference and current target, reducing the uncertainty of mosquito locations with high density.

## Materials and methods

### Study area

The trial took place in several districts of Ghana and Mozambique between September 2022 and August 2024. These districts were not selected to compare the performance of the adaptive design across different malaria transmission intensities; rather, they were chosen to enable a direct comparison with routine surveillance systems. Accordingly, we included only districts with governmental routine sentinel sites operating throughout the entire trial period and designated as high-priority areas by the National Malaria Control Programmes.

#### Mozambique

Malaria in Mozambique is endemic throughout the country, ranging from hyperendemic zones along the coast to mesoendemic zones in the inland flatlands and some hypoendemic zones in the inland highlands. Mozambique is divided into three regions based on malaria prevalence. The northern region has a prevalence ranging from 33.8% to 54.7%, central region from 10.2% to 32% and southern region from 0.0% to 15.8%.^13^ The major malaria vectors in Mozambique are *Anopheles gambiae s.s*., known to be prevalent in the northern and central regions, *An. arabiensis*, most prevalent in the central and south and *An. funestus s.s*., considered widely present in the coastal areas of the country.^13^ The northern region includes the provinces of Niassa, Cabo Delgado and Nampula; the central region includes the provinces of Tete, Zambézia, Manica and Sofala; and the southern region includes the provinces of Inhambane, Gaza, Maputo and Maputo City. Typically, Mozambique has a tropical climate with two distinct seasons: a hot, rainy and humid season from November to March and a dry and cool season from April to October, although there are variations depending on the region and altitude.^14^ Malaria transmission is higher in the hot and humid season due to the abundance of water and vegetation, which favours breeding of *Anopheles* mosquitoes.^14^ *Plasmodium falciparum* accounts for 99.7% of all malaria infections.^13^ Mozambique contributes around 4% of the global malaria case burden.^1^ The trial took place in two districts in the province of Niassa (Cuamba and Mandimba) and one in the province of Zambezia (Morrumbala).

In Niassa, the rainy season extends from November to April. Most of the population works in farming activities such as food crop production, livestock rearing and the selling of charcoal. There is also non-farm employment at a very small scale.^15^

Zambezia has a considerable forest inland, and much of the coast consists of mangrove swamps.^16^ Zambezia province is characterised by a seasonal pattern of rainfall from October to June, and malaria is perennial with transmission peaking during the rainy season. The economy of Zambezia is dominated by subsistence agriculture (88% of the population works in agriculture).^17^

#### Ghana

Ghana has a warm tropical climate with two distinct seasons (dry and wet). The South of Ghana is warm and humid, whereas the North of Ghana is hot and dry. The rainy season runs from March to November in the North and from July to September in the South. The estimated population average malaria parasite prevalence is between 2% and 15% among children 6–59 months of age.^18^ *P. falciparum* accounts for more than 95% of infections.

The trial was conducted in five districts across three regions of Ghana: Ga West and Ga South in Greater Accra Region, Ketu North and Ketu South in Volta Region, and Suaman in Western North Region.

The Greater Accra Region is the most populous region in Ghana, home to around 5.5 million people.^19^ The Greater Accra Region is in the coastal climatic zone, with high humidity and average daily temperatures above 27°C. Accra is a centre for manufacturing, marketing, finance, insurance and transportation.^20^

The Volta Region of Ghana spans from the coastal climatic zone in the South to the forest climatic zone in the North. 72% of the population live in rural areas with agriculture, hunting, forestry and fishing as the main industries.^21^

The Western-North region of Ghana falls within the forest climatic zone.^22^ Farming constitutes the major occupation for livelihood. Others are miners, civil servants and traders.

### Environmental data

We used open and freely available remote sensing data from several sources to model the data from Routine and Adaptive designs. Remote sensing data included land cover, climate and topography from the US National Aeronautics and Space Administration Earth Observing System Data and Information System Land Processes Distributed Active Archive Center (EOSDIS LP DAAC), the European Space Agency and the US Geological Survey (full details provided in Table S1 in online supplemental file l). All environmental variables were used at their original spatial and temporal resolution (no downscaling or upscaling).

### Trapping methods

The trapping methods employed in the adaptive design replicated those used in routine surveillance, over which the authors had no control. The effects of different trapping methods on mosquito counts were accounted for in the spatiotemporal model as a covariate.

In Mozambique, adaptive surveillance design was conducted between September 2022 and August 2024 in parallel to the standard routine surveillance (see also Table S2 in online supplemental file l). During the first year (October 2022 to September 2023), human landing catches (HLCs) were conducted hourly for two consecutive nights both indoors and outdoors per house per month in Cuamba and Mandimba. Instead of HLCs, and only during the first year, Centers for Disease Control and Prevention (CDC) light traps (CDC LTs; Model 512, John W Hock, Florida, USA) were used in Morrumbala district. Traps were installed indoors near an occupied bed and outdoors in combination with a tent (CDC-LT+Tent) occupied by a study volunteer and were operated for three consecutive nights per house each month. A total of 12 houses was selected in Mandimba and 9 in Cuamba. In Morrumbala district, 12 households were used for outdoor CDC-LT+Tent collections, and an additional 12 houses for indoor resting collections using both Prokopack aspirators (12 V; Model 1419, John W Hock) and CDC light traps.

During the second year (November 2023 to August 2024), HLCs were conducted under the same previous year’s protocol in 9 houses in Cuamba and 12 houses in Mandimba. HLCs were also employed in 12 houses in Morrumbala district. In the latter, differently from the previous year, CDC LTs without tents were used in 12 houses indoors only, also operated for three consecutive nights per house per month. Resting mosquitoes were sampled indoors once per house per month using Prokopack aspirators in an additional 12 houses. Therefore, in Morrumbala district, a total of 36 houses were surveyed monthly.

In Ghana, HLCs were conducted hourly both indoors and outdoors for four consecutive nights per month between July 2023 and July 2024. In all districts, eight houses were used for the collection with two houses being visited each night. To sample mosquitoes that were resting inside houses, pyrethrum spray catches (PSCs) were conducted indoors in the morning in 20 rooms per site per month, equivalent to 5 rooms per site for four mornings. Rooms selected for PSCs were those in which people slept the previous night and were different from the rooms used for HLCs.

**Figure 1:**
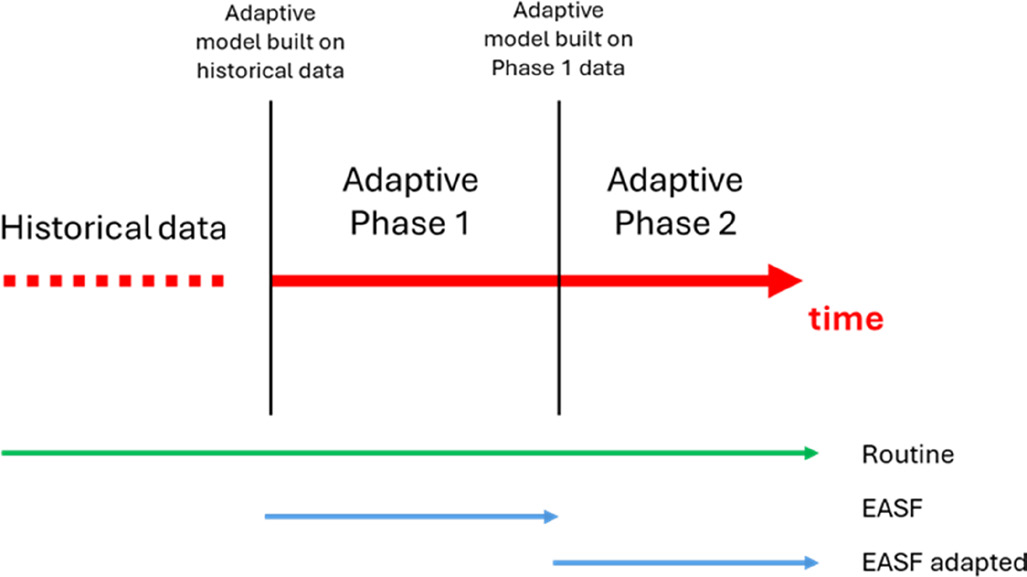
Schematic timeline for Adaptive (EASF) and Routine surveillance designs. EASF, Entomological Adaptive Surveillance Framework.

Concurrently with the Adaptive design (figure 1), Routine mosquito trapping was conducted at fixed sentinel sites as part of the National Malaria Control Programme in both countries. Routine trapping methods were the same as those used in the Adaptive design.

### Adaptive surveillance

Considering an unobserved spatial process (to distinguish it from mosquito counts or any other directly observed vector indicator), representing the geographical variation of the mosquito indicator, $S=\left\{S\left(x\right):x\in D\subset R^{2}\right\}$, where *x* is the location and *D* is the study area. The collection of all the sampling locations, *x*, is the surveillance design and can be written as *X* = (*x*_1_, …, *x_n_*). In adaptive spatial sampling designs, the surveillance design *X* and the underlying spatial process *S* are conditionally independent given Y, the set of observations (eg, mosquito counts), where *Y = (Y_1_, …, Y_n_*). In fact, let *X_0_* be the initial surveillance design, from which *Y_0_* is the resulting measurement data and *X_1_* the set of additional sampling locations (selected adaptively), from which measurement data *Y_1_* is collected; then:

$X=X_{0}\cup X_{1}$ is the adapted surveillance design and,

$Y=(Y_{0},Y_{1})$ is the overall collected measurement data.

The likelihood for the complete dataset is given by:


$$[X,Y]=\int_{s}\;[X,Y,S]dS$$

where the [ ] denotes ‘the distribution of’. After standard factorisation for multivariate distribution and subsequent simplifications, the likelihood for the complete dataset takes the form of:


$$\left[X,Y\right]=\left[X|Y_{0}\right][Y|X]$$

which shows that *X* and *S* are conditionally independent given *Y*, because only *Y* is sufficient to describe the relationship between *Y* and *X*, independent of *S*.^8^

The implementation of the adaptive surveillance design requires at least three elements: (1) an initial model to fit the data; (2) a target function; and (3) a rule (batch size and prioritisation) for selecting new locations to be added adaptively.^23^

The Adaptive design, hereafter referred to as the ‘Entomological Adaptive Surveillance Framework’ (EASF), was initially implemented using historical data, with differences in trapping methods, climate and ecological conditions included as covariates. This initial sampling design was subsequently refined (based on the target function), as new data became available. This iterative process substantially reduces the risk that early design choices, often made under uncertainty, will lock the system into a biased or suboptimal framework. The target function was then evaluated after 12 months in Mozambique and 6 months in Ghana (the Ghana trial started ten months later).

#### Statistical model

The spatiotemporal model with nugget effects,^24^ used for modelling the observed mosquito counts (*Y*) is:


$$\log\left(Y_{j}\right)=\mu_{j}+\epsilon_{j}$$

$$\varepsilon_{j}\sim Normal(0,\sigma_{\epsilon }^{2})$$

where *ϵ* is the pure error term (white noise or nugget effect), assumed to follow an independent zero mean normal distribution with variance *σ^2^; μ* is the mean process of the model; and *j* is the spatiotemporal location (at a site *x* and time point *t*). We used log-transformed counts to correct for heavy-tailed distribution (which is the case in the context of mosquito populations). In addition, the lognormal distribution allows for a simplified likelihood structure, particularly when partitioning the variance among different model components within a Bayesian framework.^25^

The spatiotemporal model has a Bayesian structure and includes both spatial and temporal correlation. The mean process *μ* is modelled as:


$$\mu_{j}=W_{j}\beta +\rho \mu_{a}+\eta_{j}$$

where **W*_j_*** is the design matrix containing the covariate values at location *j*, and *β* is a vector of unknown regression coefficients; *ρ* is an autoregressive temporal correlation parameter representing the strength of temporal dependence of mosquito counts between successive months; and *ƞ* is a spatiotemporal random effect that follows a Gaussian process with mean zero and covariance function of a Matern class correlation function. The spatiotemporal random effects *η*(t=1), *η*(t=2), …, *η*(t=T) are assumed to be independent in time. The subscript *a* indicates the spatiotemporal location for site *x* and time point *t*-1, distinguishing it from *j* = (*x,t*). The model parameters are estimated using Markov Chain Monte Carlo methods (full inference described in the study by Sahu^24^).

#### Target function

For this field trial, the EASF target is the identification of locations with higher average of mosquito abundance for *Anopheles spp*. in Ghana and *An. gambiae* and *An. funestus* (accounted together) in Mozambique, as well as higher uncertainty in terms of average prediction error. In this way, we aimed to improve the accuracy of hotspot delineation, that is, identifying areas with the highest probability to collect mosquitoes.

The target function is based on the peaks-over-threshold theory, in which exceedances are assumed to follow a generalised Pareto distribution in both space and time.^26^ The probability that the predicted value y*, mosquito counts or densities, is larger than a prespecified threshold (ie, the median value of y*) denoted by *h_y_*, is given by:


$$F\left(y^{*}\right)=Prob\left(h_{y}§amp;lt;y^{*}\right)=exp\left\{-\lambda \left[1-G\left(y^{*}\right)\right]\right\}$$

where the exceedance distribution G(y*) is defined as:


$$G\left(y^{*}\right)=1-{\left[1-k\left(\frac{y^{*}-h_{y}}{\tau }\right)\right]}^{\frac{1}{k}},k\neq 0,y^{*}§amp;gt;h_{y}$$

where *G*() is the cumulative distribution function of the generalised Pareto distribution, with *k* and *τ* the shape and scale parameters, respectively. The parameter *λ* is the Poisson rate, which quantifies the expected number and variance of exceedance occurrences.

The same function *F*() can be applied to the uncertainty estimates, *q*, associated with y*. The similarity between exceedances of F(y*) and *F(q*) across spatiotemporal locations *j* is tested using Bayesian factor analysis, an alternative to asymptotic likelihood ratio test. The Bayes factor assesses the plausibility of the two different models F(y*) and *F(q*), and it is defined as the ratio of their marginal likelihoods. Values between 0 and 2 indicate no substantial difference.^27^

The minimisation of the Bayes factor is the target function used in the adaptive surveillance design, which aims to identify the spatial and temporal allocation of surveillance sites that minimise the errors of the statistical model while targeting areas where predicted mosquito abundance (or density) and associated uncertainties show similar spatiotemporal exceedance patterns (or in other words where counts are both high and uncertain). This allows the ascertainment of potential hotspots.

To optimise the target function and determine the optimal number of spatial locations and temporal trapping frequency, we developed a simulation annealing algorithm applied to the postmodel inference results. This is a common technique in geostatistical adaptive surveillance design.^28^ The algorithm (box 1) is repeated for a set of batches of different sizes, expressed as vector *L,* with, for example, a set of *v* values: *L* = (*l_1_*=15, *l*_2_=20, …, *l*_v_=*M*) new locations, where *M* is the maximum number of new locations considered. For large areas and temporally frequent surveillance, batch sizes are typically large (>100 new locations per iteration) to adequately explore the different spatiotemporal adaptive configurations. After the algorithm is executed across all the batch sizes in *L*, the optimal batch size and the relative surveillance design is identified as the one that yields the lowest cross-validation predictive error, recalculated under the assumption that the values in l were observed rather than estimated. The minimum temporal resolution is then derived as the minimum number of repeated observations required at each new location in the optimal batch.

Box 1 **Adaptive framework of the EASF.**

**Algorithm: simulated annealing for adaptive site selection**

Input: consider one of the location batches in *L*, eg, the first one: *l*_1_

Step 1 (weight assignment): assign normalised weights to prioritise uncertain hotspot areas. Each weight combines the scaled (range 0 and 1) estimated counts and uncertainties:

$${\text{weight}}_{1}=\frac{1}{2}\left({\bar{y}}_{1}+{\bar{q}}_{1}\right);{\bar{y}}_{1}=\frac{{\hat{y}}_{1}-\min(\hat{y})}{\max(\hat{y})-\min(\hat{y})};\;{\bar{q}}_{1}=\frac{{\hat{q}}_{1}-\min(\hat{q})}{\max(\hat{q})-\min(\hat{q})}$$

Step 2 (initialisation): let *B*_*0*_ be the initial Bayes factor value; eg, based on the current surveillance design.

Step 3 (iterative allocation):

**for***i* in 1, … **do**

- allocate the new locations *l*_1_ from the set *L* in the study area (*l*_1,*i*_) through weighted random sampling based on weight_1_
- extract expected mosquito counts and standard deviations at *l*_1,*i*_ (stored as *Y*_1,*i*_)
- compute exceedance probabilities F(y*)_*i*_ and *F(q)*_*i*_ for the updated dataset *Y*_1,*i*_ U *Y* dataset
- generate a candidate configuration *B*_*i*_
- compute the acceptance probability, *z*_*i*_ using the simulated annealing criterion

$z_{i}=\exp\left(-\frac{f(B_{i})-f(B_{0})}{C_{i}}\right)$

- if *z*_*i*_ >1 or *z*_*i*_ > R_*i*_, then set *B*_0_ = *B*_*i*_ and accept the candidate configuration *l*_1,*i*_
- otherwise: *B*_0_ stays unchanged and retain current configuration *l*_1,*i*-1_
- continue until *B*_0_ remains unchanged for N consecutive iterations (stopping criterion).

**End of for**

Step 4 (repetition): repeat steps 1 to 3 with each remaining batch *l* in *L.*

B, Bayesian factor; C, cooling coefficient (or cooling schedule); EASF, Entomological Adaptive Surveillance Framework; N, maximum number of repetitions without acceptance necessary to stop the algorithm; R_*i*_, a randomly generated number from a uniform distribution between 0 and 1; Y, observed data at surveyed locations.

#### Prioritising locations

Once the optimal batch size and the corresponding spatiotemporal surveillance design is generated, locations are prioritised based on their capacity to improve the accuracy of hotspot delineation (largest improvement=highest priority). This priority list was provided to the National Malaria Control Programmes in each country. The actual number of prioritised sampling locations was further downscaled to allow feasibility of the sampling design.

The feasibility of collecting data at each prioritised site was assessed using a combination of satellite imagery (Google Earth) and ground truthing. Factors considered in feasibility assessment included accessibility of the EASF generated sampling locations during all seasons, available budget, distance between locations (locations that were far apart and away were deemed not feasible for quality and safety in night time monitoring of entomology activities), available human capacity present at the National Malaria Control Programmes to run the activities alongside the routine and community consent.

EASF surveillance locations are considered as neighbourhoods of 1 km^2^, which corresponds to the coarsest spatial resolution of the environmental variables (Table S1 in online supplemental file l). In Mozambique, surveillance houses were then selected ∼70–90 m from each other within the final prioritised surveillance locations. In Ghana, the house selection process involved dividing the target area/community into four quadrants. From each quadrant, two houses were randomly selected, yielding a total of eight houses.

### Statistical analyses on surveillance designs

#### Objective one: comparison between the EASF and Routine surveillance designs

We compared the EASF and Routine surveillance in terms of: average mosquitoes collected per method of trapping; predictive capacity of the collected data to evaluate the spatial and temporal bias in the data; redundancy of the information gathered from each surveillance location through identification of obsolete surveillance locations.

The sampling effort is defined as the total number of house visits conducted for mosquito collection under each surveillance design and trapping method.

To assess predictive accuracy of the data from the EASF and Routine surveillance designs, we applied a negative binomial log-linear regression model using the environmental variables (from Table S1 in online supplemental file l) as fixed effects, and including a spatiotemporal Gaussian random effect term to account for extra variations over space and time. For each surveillance design, we randomly selected a subset of data to estimate the model and used the obtained model to make predictions at the spatiotemporal locations of the other surveillance design. In other words, the random subset of the EASF data was used to fit a negative binomial log-linear regression model, which was then used to predict data values at the spatiotemporal locations of the Routine design, and vice-versa. We measured prediction accuracy using the root mean square standardised prediction error (RMSSPE), where lower values indicate better predictive performance. To control for sampling effort (since EASF sampling effort was much larger than Routine), we randomly selected subsets of data from the EASF to match the number of locations for each month surveyed in the Routine design. For example, if in January 2024 the Routine design surveyed three locations, three locations in January 2024 were randomly selected from the EASF design to match the sampling effort of the Routine design; and so on for all the months and years surveyed in the Routine design.^29^ This random selection process was repeated 499 times, and the results were averaged.

Obsolete locations are defined as sites where the information collected does not influence the quality of the model inference and estimation and therefore these locations are unnecessary for improving the statistical model adopted in adaptive surveillance. To identify obsolete surveillance locations, we evaluated the effect of removing one or more locations, at any point in time after the first 5 months of surveillance, on the log-likelihood of the negative binomial model. Log-likelihood ratio was compared against a critical value from a χ² distribution with degrees of freedom adjusted for sample size differences.^30^ Locations (in space and time) associated with models with log-likelihood ratio lower than the critical value were classified as obsolete.

#### Objective two: internal evaluation of the EASF and Routine surveillance designs

In objective two, the initial surveillance, designed by the adaptive method applied to historical data (phase 1), is compared with the data obtained from the subsequent surveillance (phase 2) (figure 1).

The evaluation of the models employed for the EASF surveillance design is based on three metrics: goodness-of-fit (through predictive model choice criterion (PMCC), see definition below), model predictive capability (RMSSPE) and the coefficient of variation (CV) of the estimates. The evaluation is based on the comparison between phase 1 and phase 2 of the EASF surveillance through PMCC and CV, and between EASF and Routine (used as benchmark) through RMSSPE. Evaluating the goodness-of-fit (PMCC) and robustness (RMSSPE) of the model from which the adaptive design originates is equivalent, in this context, to evaluating the effect of the adaptive surveillance on the accuracy of the model parameters.

Goodness-of-fit was evaluated by employing a Bayesian model choice criterion known as PMCC.^31^ Mathematically, it is defined as:


$$PMCC=\sum_{j=1}^{n}(y_{j}^{*}-y_{j}{)}^{2}+\sum_{j=1}^{n}Var\;(y_{j}^{*})$$

where the first term in the right-hand side represents the expected squared error (goodness of fit), while the second term is a penalty term for model uncertainty. Here, y* is the prediction at the spatiotemporal location *j*, while *y* is the observed (real) value. A model with the smallest PMCC is considered a better model.

For the model predictive capability, we follow the same procedure applied in Objective one. A negative binomial log-linear regression model was inferred for each surveillance design, but instead of using the model to predict locations at the other surveillance design, the model was used to predict locations at the same surveillance design. In other words, a subset of the data from one surveillance design was used to fit a negative binomial log-linear regression model, which was used to predict values at the left-out spatiotemporal locations. As a comparator, this was done for each of the EASF and Routine datasets, and as described above, the Routine dataset was compared with subsets of the EASF dataset to maintain and compare the same sampling effort. Predictive accuracy was assessed through RMSSPE.

To determine if model robustness improved between phase 1 and phase 2 of EASF surveillance, the mean and SD of the model predictions were compared through the CV, a dimensionless measure of relative variability scaled to the mean. A value close to one means clumping in the data and not overdispersed (coherent with the parameterisation of a Poisson distribution where the mean parameter is at the same time the expected mean and variance of the distribution).^32^ To test the difference between CVs, we used the modified signed-likelihood ratio test for equality of CVs.^33^

## Results

Mosquito collection in Mozambique under the EASF and Routine designs took place between September 2022 and August 2023 (year 1) and from November 2023 to August 2024 (year 2); and in Ghana between July 2023 and July 2024 (year 1) (Figure S1 in online supplemental file l). Different trapping methods were employed in both countries (Table S2 in online supplemental file l).

In table 1, the Bayesian estimates (posterior means) and their corresponding 95% credible intervals for the coefficients of all ecological predictors included in the country-based models (using the entire data not a subset) are shown. Only two predictors were consistently significant in the three models (from merged EASF and Routine datasets, EASF dataset only and Routine dataset only); the daytime land surface temperature and the nighttime land surface temperature. For the latter, all models found a positive association, with increases in nighttime land surface temperature associated with increases in mosquito counts. For daytime land surface temperature, the association with mosquito counts is positive for Ghana in the Routine models and negative for both countries within the EASF model. compared with the Routine model, the EASF model for both Ghana and Mozambique has found a larger number of statistically significant predictors (eight for Ghana and three for Mozambique). The inclusion of trapping method as a covariate did not significantly improve the country-level spatiotemporal model.

**Table 1:** Coefficients for the negative binomial log-linear regression model applied to the full data collected in the trial (EASF and Routine), and for each surveillance design

	EASF+routine		Routine		EASF	
	Country	Estimated coefficient and 95% credible intervals	Country	Estimated coefficient and 95% credible intervals	Country	Estimated coefficient and 95% credible intervals
Elevation	**MZB**	**0.36** (**0.09 to 0.63**)	NS	NS	**GHN**	**−0.48 (−0.57 to −0.39**)
Pop. density	NS	NS	NS	NS	NS	NS
Land cover: cropland	NS	NS	**MZB**	**1.57** (**0.00 to 3.24**)	**GHN**	**0.25** (**0.17 to 0.32**)
Land cover: grassland	NS	NS	NS	NS	NS	NS
Land cover: shrubs	NS	NS	NS	NS	**MZB**	**−0.82 (−1.62 to −0.02**)
Land cover: trees	NS	NS	NS	NS	**GHN**	**0.28** (**0.10 to 0.47**)
Land cover: vegetation aquatic or regularly flooded	NS	NS	NS	NS	**GHN**	**−0.36 (−0.56 to −0.17**)
LST_Day_1 km	**MZB** **GHN**	**−0.52 (−0.84 to −0.20**) **−0.32 (−0.42 to −0.22**)	**GHN**	**0.38** (**0.06 to 0.70**)	**MZB** **GHN**	**−0.36 (−0.70 to −0.02**) **−0.27 (−0.32 to −0.22**)
LST_Night_1 km	**MZB**	**0.27** (**0.00 to 0.54**)	**GHN**	**0.34** (**0.02 to 0.66**)	**MZB** **GHN**	**0.32** (**0.02 to 0.63**) **0.17** (**0.12 to 0.21**)
Gpp_500 m	NS	NS	NS	NS	**GHN**	**11.31** (**6.69 to 15.93**)
PsnNet_500 m	NS	NS	NS	NS	**GHN**	**−11.49 (−16.12 to −6.86**)
sur_refl_b07	**GHN**	**−0.19 (−0.33 to −0.05**)	NS	NS	NS	NS
EVI_16_days	NS	NS	NS	NS	**GHN**	**0.09** (**0.00 to 0.19**)
NDVI_16_days	NS	NS	NS	NS	**GHN**	**0.19** (**0.08 to 0.31**)

Only statistically significant coefficients per country of surveillance are shown (GHN: Ghana; MZB: Mozambique).

EASF, Entomological Adaptive Surveillance Framework; EVI, Enhanced Vegetation Index; GPP, Gross Primary Production; LST, land surface temperature; NDVI, Normalised Difference Vegetation Index; NS, not significant; PsnNet, net photosynthesis; sur_refl, surface reflectance.

### Objective one: comparison EASF versus Routine

Summing all trapping methods, in Mozambique, an average of 8.59 mosquitoes were collected using the EASF design, with a CV of 2.13. In contrast, the Routine design yielded an average of 2.85 mosquitoes with a CV of 2.24. In Ghana, the EASF design collected an overall average of 4.35 mosquitoes with a CV of 1.88, while the Routine design collected an average of 3.61 mosquitoes with a CV of 2.27.

The EASF design collected significantly more mosquitoes than the Routine design for CDC (with CDC LTs, and with CDC LTs+tent) and HLCs in Mozambique and PSCs in Ghana. However, there was no significant difference between the two designs when using the Prokopack method in Mozambique or the HLC method in Ghana (table 2).

**Table 2:** Total and average number of *Anopheles* mosquitoes collected in Ghana and Mozambique by trapping method and surveillance design

Country	Species	Collection method	Collected mosquitoes: totals and (average per trap event)		P value
			Routine	EASF	
Mozambique	*Anopheles gambiae*	HLCs	31 (0.69)	**2060** (**5.02**)	**0.014**
		CDC*	102 (2.37)	667 (3.30)	0.832
		Prokopack	4 (0.06)	14 (0.06)	0.318
	*Anopheles funestus*	HLCs	32 (0.71)	905 (2.21)	0.059
		CDC*	52 (1.21)	**789** (**3.91**)	**0.037**
		Prokopack	10 (0.14)	34 (0.15)	0.870
	*Anopheles maculipalpis*	HLCs	0 (0)	15 (0.04)	0.227
		CDC*	4 (0.09)	208 (1.03)	0.101
		Prokopack	0 (0)	0 (0)	NA
	*Anopheles tenebrosus*	HLCs	13 (0.29)	149 (0.36)	0.921
		CDC*	0 (0)	2 (0.01)	0.518
		Prokopack	1 (0.01)	0 (0)	0.073
	*Anopheles coustani*	HLCs	0 (0)	19 (0.05)	0.227
		CDC*	1 (0.02)	83 (0.41)	0.180
		Prokopack	0 (0)	0 (0)	NA
	*Anopheles other species*	HLCs	8 (0.18)	230 (0.56)	0.190
		CDC*	8 (0.19)	130 (0.64)	0.191
		Prokopack	0 (0)	1 (0.004)	0.585
	*Total Anopheles*	HLCs	84 (1.87)	**3378** (**8.24**)	**<0.001**
		CDC*	167 (3.88)	**1879** (**9.30**)	**0.007**
		Prokopack	15 (0.21)	49 (0.22)	0.784
Ghana	*Anopheles spp*.	HLCs	1961 (8.60)	9151 (9.66)	0.201
		PSCs	101 (0.29)	**3885** (**1.89**)	**< 0.001**

Differences between surveillance designs are tested through the Wilcoxon test, from which the p values are reported in the last column (in bold values below a 5% significance level).

* CDC refers to CDC LTs and CDC LTs+tents.

CDC LTs, Centers for Disease Control and Prevention light traps; HLCs, human landing catches; NA, not assessed; PSCs, pyrethrum spray catches.

The comparison between phase 1 and phase 2 of the EASF design resulted in different outcomes in the two countries. In Ghana, phase 2 allowed for the capture of more mosquitoes with a larger uncertainty (SD) than in phase 1; but in Mozambique, a lower number of mosquitoes was collected in phase 2 than in phase 1, with lower uncertainty (table 3). This means that the Mozambique model to identify hotspots for phase 2 overpredicted the hotspots. It is interesting that the more accurate model of Ghana was built with 20% additional mosquitoes compared with the number of mosquitoes in the Mozambique model.

**Table 3:** Comparison of the EASF design between phase 1 and phase 2 of the adaptive design

Country	Collection method	Mosquito counts (mean)		P value
		Phase 1	Phase 2	
Mozambique	HLCs	2665 (11.7)	713 (3.90)	<0.001
	CDC LTs	1689 (15.9)	190 (1.98)	<0.001
	Prokopack	28 (0.21)	21 (0.22)	0.374
Ghana	HLCs	3902 (9.14)	5249 (10.10)	0.414
	PSCs	1375 (1.57)	2510 (2.14)	0.495

CDC LTs, Centers for Disease Control and Prevention light traps; EASF, Entomological Adaptive Surveillance Framework; HLCs, human landing catches; PSCs, pyrethrum spray catches.

In terms of the capacity of the EASF model to predict Routine data and vice-versa, in Mozambique, EASF has a lower average error (therefore greater prediction capacity, see Table S3 in online supplemental file l) with 99.6% of the RMSSPE values for EASF data predicting Routine locations smaller than or equal to the average RMSSPE for Routine data predicting EASF locations. Conversely, only 4% of the RMSSPE values for Routine data predicting EASF locations were smaller than the average RMSSPE for EASF data predicting Routine locations. This indicates that the model generated from EASF data had a much lower prediction error when predicting data from Routine locations, considering the overall distribution of the posterior predictions, showing that EASF data has better predictive power than the Routine data in Mozambique. However, in Ghana, a lower predictive power than Mozambique has been found with 73.1% of the RMSSPE values for EASF data predicting Routine locations smaller than or equal to the average RMSSPE for Routine data predicting EASF locations.

Regarding obsolete locations (ie, locations with no significant contribution to the model inference and estimation), the Routine design had a higher proportion of obsolete surveillance locations compared with the EASF design in both countries. Around a third of the locations were found obsolete in the Routine design compared with between 5%–16% in the EASF design (Table S4 in online supplemental file l).

### Objective two: internal evaluation of the EASF and Routine surveillance designs

In Ghana, the data collected from the EASF design produced a more robust model than the Routine data, although the opposite was found in Mozambique (Table S5 in online supplemental file l). CIs were not calculated for the Routine data model, since all the data are used and not subsets as for the EASF dataset.

A negative value of PMCC difference indicates a reduction during the adaptive process, meaning a better model in terms of predictive power. The model improved (decrease in PMCC) during the adaptive phase in Mozambique but not in Ghana, although in both countries the EASF predictions decreased in variation (negative CV differences for both countries) (Table S6 in online supplemental file l).

## Discussions

The Entomological Adaptive Surveillance Framework (EASF) was developed to overcome the limitations of long-term surveillance at fixed sentinel sites.^34^ Over time, these sites may become obsolete if they no longer provide new, meaningful insights into *Anopheles* mosquito populations. Prolonged monitoring at the same locations may fail to capture the spatial variability of mosquito distribution, leading to an over-representation of certain areas while missing emerging hotspots.^35^ Additionally, if mosquito population dynamics remain stable over multiple surveillance periods, continued data collection may yield diminishing returns.

In our comparison between the EASF and Routine designs, we have shown that most of the metrics evaluated in this study were superior in the EASF design. In Ghana and Mozambique, collections of mosquitoes (summing all trapping methods) were greater at EASF sites than Routine sites. Our results show that the EASF model had a stronger ecological signal, as per number of significant predictors. Furthermore, we found an increased average number of collected mosquitoes accompanied by a reduction of CV by using the EASF in comparison to the Routine. In addition, the final EASF predictions had a significantly lower CV than the initial EASF predictions, indicating more stable and robust models (model’s estimates are more consistent and less prone to large fluctuations). That, combined with the reduction of CV in the real data, confirms the efficacy of the adaptive design to reduce the uncertainty in both collected and estimated data.^36 37^ When considering each of the trapping methods individually, the collections were not statistically significantly different for HLC collections in Ghana and Prokopack in Mozambique.

In both countries, the EASF model was more robust (better at predicting EASF locations), and generalisable (better at predicting Routine locations, although not significantly different in Mozambique) than the Routine model. In Mozambique, the EASF underperformed in the predictive accuracy over its own locations, in comparison to the Routine design. This may be due to the existing trade-off between inferential and estimation accuracy, where minimising prediction errors requires penalising the goodness of fit—or reducing overfitting (also known as accuracy paradox),^38^ especially when the area encompassed by EASF locations is much larger than the one of Routine surveillance. To reduce this issue, future adaptive designs should consider the allocation of new batches that focus on the general improvement of the inference in addition to the hotspot delineation goal.^8^

In the evaluation of the ability of the adaptive design to improve the target function (in statistical terms we refer ‘to maximise’ the target function), while one full year of phase 1 data collection in Mozambique allowed improvements in the adaptive design’s model (in terms of PMCC), 6 months were not sufficient in Ghana. One of the reasons is that phase 1 in Ghana was designed on historical data with uncertain sampling effort, unknown mixing of trapping collection and mosquito species, and with zero-deflated (completely missing) data which is likely to have generated inferential modelling biases.^39^ Further implementation of joint modelling for missing-zeroes data^40^ in an adaptive framework is under development.

For the evaluation of redundant information, the proportion of locations classified as ‘obsolete’ in the EASF design was two to three times lower than the proportion of obsolete locations in the Routine design. This indicates that adaptive surveillance was more efficient in generating informative data than routine surveillance.

It is important to stress that during adaptive surveillance, an improvement of the target function may not correspond to achieving the desired target (eg, a specific accuracy in mosquito collection estimation). Defining how many adaptive iterations and when these occur is one of the main challenges in adaptive design. As in most simulated adaptive studies, the target function is never maximised after the first adaptation of the surveillance design (adding or removing sites).^41^ In addition, the time for adaptive surveillance to reach its goal depends on the complexity of the target function. In a recent study on adaptive mosquito surveillance in Benin, the authors highlighted the difficulty of satisfying a multigoal criteria with a single round of adaptive locations.^23^ That said, it is our opinion that the main operational quality of an adaptive design is not reaching a specific objective, but adapting the current surveillance towards its goal(s), being flexible in case of new findings and satisfying budgets. In this context, the question about the number of adaptive iterations is less important than how we can maintain cost-efficient surveillance programmes.

In this work, we have targeted uncertain hotspots. However, the use of the adaptive framework can have other goals: for example, finding locations that can best represent the effect of interventions or particular climate conditions. If a National Malaria Control Programme wants to evaluate the impact of an intervention over time, the optimisation criteria should return those sites with high uncertainty between intervention and abundance. If maximising mosquito collections while reducing uncertainty is still the goal, this could also be combined with additional criteria like sample size (eg, for genetic analyses), accessibility, etc,^42^ to further refine the surveyed-to-be sites. A surveillance programme could decide what these additional criteria should be, including being appropriate for long-term monitoring, although multicriteria approaches can require longer surveillance campaigns,^23^ in which case prioritisation of the target should be considered to reduce the length of the surveillance. In addition, the frequency with which a design goes through the adaptive approach, and the geographical scale at which the model is run, can be decided a priori based on the process to monitor^43^ and similarities between current areas surveyed and areas planned for surveillance.^44^

Adaptive designs should start with the questions: what is the value of better data? And at what thresholds of an output may decisions change? As expected, better data should lead to better decisions, and we can simulate these decisions and their potential impact by determining whether the EASF set of data would have resulted in a different outcome than the standard surveillance data. For current surveillances, an adaptive design may help in answering the following questions: is my current surveillance approach cost-effective? Is my current surveillance approach describing the target in the most accurate way? How can I change or modify my current surveillance approach to include new objectives? How can I discriminate between large-scale effects (climate) and local effects (eg, behavioural changes)? The combination of adaptive and routine designs can improve the accuracy while keeping the number of surveillance locations fixed in order to comply with limited budgets or WHO standards^45^; or keep the current accuracy level while reducing the surveillance locations; or to reach faster detection of changes while fixing accuracy and/or number of surveillance locations/times. We understand that some surveillance campaigns have a long longitudinal record and changing it may disrupt the valuable information gained so far. However, the decision to not modify current surveillance systems must also come from an evidence-based approach such as a model-based approach. In this context, mathematical or statistical challenges/capacity limitations should not only be a barrier for adaptive surveillance, but also to run routine surveillance efficiently.^46^ Adaptive responses can help inform best practices to mitigate public health impacts from the emergence, re-emergence and spread of disease.

### Limitations

For a large dataset (or areas) there are potentially a very large number of combinations of larger than average expected mosquito counts and larger than average expected uncertainties to compute for a given set of locations—meaning increased length and complexity in the computations. In addition, focusing solely on improving the accuracy of the hotspots (the places with large counts and large uncertainties) may cause the surveillance design to minimise the target function towards a local optimum instead of the global optimum.^47^ This is the case when adding locations in areas with large uncertainties causes other areas to increase their uncertainties, becoming hotspots themselves. Therefore, studies are necessary to evaluate designs that allocate a number of sites outside the hotspot areas in order to control for the overall uncertainty while improving local uncertainty.

Mosquito hotspot identification is only one objective of entomological surveillance. Understanding vector species distribution and composition, monitoring insecticide resistance and characterising vector bionomics are equally important, as these factors directly influence intervention choice and programme design, particularly when applying tools such as the Entomological Surveillance Planning Tool.^11 48^

The EASF was not evaluated across areas with different malaria transmission intensities, which limits our ability to assess its performance across diverse epidemiological contexts. In addition, this study did not consider interventions implemented and their coverage. However, including interventions as time-correlation or space-correlation or time-and-space correlation and trends disruptor may have improved the inference in the model and reduced the target uncertainties.^49^

The representativeness of remote sensing data for local information, instead of weather station data, is a long-established and well-established debate.^50^ Our decision to use only remote sensing data, as well as excluding household-level data, is due to the need to predict over the same set of variables outside the study area, avoiding uncertainty due to the sparsity of weather station sites and the missingness of household information data for areas not surveyed or where this information is not available.

As described in the trapping methods, the adaptive design replicated the trapping protocol used at routine sites and implemented by the National Malaria Control Programme. However, we acknowledge that employing different trapping methods over short periods may influence analyses that are sensitive to environmental conditions, human behaviour or other systematic biases. Nevertheless, conducting trapping for as few as two nights is a common practice in malaria vector surveillance and in comparative evaluations of trapping methods.^51 52^ Finally, uncertainty can be reduced only to a particular point, unless all the risk factors are accounted for by the model (net campaigns, household infrastructure changes, etc) or when there is a strong relationship between the ‘unknown factors’ and the spatial and temporal random effects.^53^ However, mapping these uncertainties can provide some clues and theories around hidden factors associated with mosquito abundances.

## Conclusions

The EASF adaptive surveillance model was employed on mosquito catches collected monthly over several nights and houses to improve the accuracy of *Anopheles* hotspot delineation. The model included a set of independent spatiotemporal ecological variables (from satellite) and random effects which account for the dependence of the data in space and time (in other words, for the fact that two close locations are likely to show similar patterns in mosquito counts than two locations far apart from each other—similarly for two locations close in time). A similar model was employed for the routine data, although not with the same set of significant independent variables. In comparison to the Routine design, the EASF required increased sampling efforts during its initial stages to identify which locations provided the best gains toward the target function, with consequent cost and logistic implications. Overall, the EASF design outperformed the Routine design in most of the metrics. However, Routine and Adaptive designs should not be considered separately, and Routine surveillance may use the learning advantages brought by the Adaptive design, such as the information quality control at the surveyed locations and quantification of reduced uncertainty. This is the most effective strategy to make surveillance an instrumental control tool.^35^

## Supplementary material

10.1136/bmjph-2025-004060 online supplemental file 1

## Data Availability

Data are available upon reasonable request.
