# Supplementary material for: Evaluation of the Entomological Adaptive Surveillance Framework for malaria vector monitoring: a comparative field trial with routine surveillance in Ghana and Mozambique
Source: BMJ Public Health. 2026 Mar 31;4(1):e004060. doi: 10.1136/bmjph-2025-004060 (PMC13052570; doi:10.1136/bmjph-2025-004060)
Supplement: online supplemental file 1 [file bmjph-4-1-s001.docx]

**Evaluation of the Entomological Adaptive Surveillance Framework for Malaria Vector Monitoring: A Comparative Field Trial with Routine Surveillance in Ghana and Mozambique**

Luigi Sedda, Abdollah Jalilian, Mercy Opiyo, Steven Gowelo, Dulcisária Jotamo Marrenjo, Christian Atta-obeng, Ernest Boampong, Samuel Kweku Oppong, Otubea Owusu-Akrofi, Edward Thomsen, Allison Tatarsky, Keziah Laurencia Malm, Baltazar Candrinho, Neil F. Lobo

**SUPPLEMENTARY INFORMATION**

| 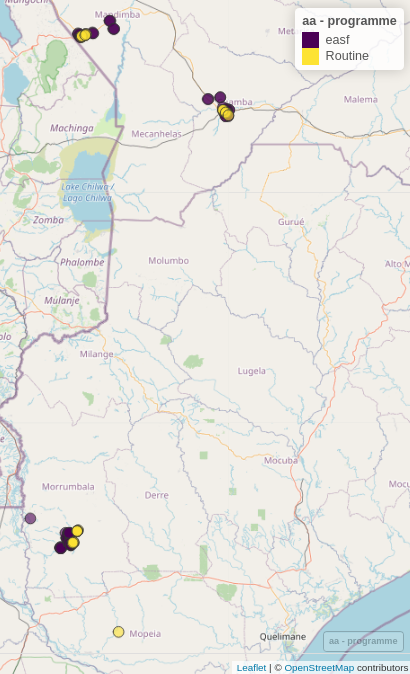 | 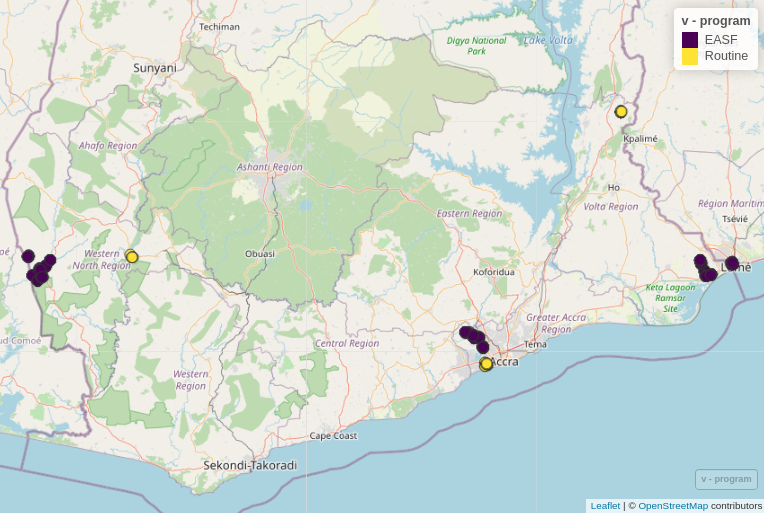 |
| --- | --- |

Figure S1. Surveillance locations for the EASF (yellow) and Routine (purple) designs in Mozambique (left) and Ghana (right).

Table S1. Environmental variables.

| **Variable** | **Code** | **Unit** | **Source and link** | **Resolution** |
| --- | --- | --- | --- | --- |
| Daytime Land Surface Temperature | LST_Day_1km | Kelvin | Moderate Resolution Imaging Spectroradiometer (MODIS)  <https://planetarycomputer.microsoft.com/dataset/group/modis> | daily  1 kilometre |
| Nighttime Land Surface Temperature | LST_Night_1km | Kelvin |  | daily  1 kilometre |
| Gross Primary Productivity | Gpp_500m | kilograms of Carbon per square meter |  | 8 days  500 meters |
| Net Photosynthesis | PsnNet_500m | kilograms of Carbon per square meter |  | 8 days  500 meters |
| Surface Spectral Reflectance for Band 7 (mid-infrared) | sur_refl_b07 | Dimensionless (reflectance values) |  | 8 days  500 meters |
| Enhanced Vegetation Index | EVI_16_days | Dimensionless |  | 16 days  250 meters |
| Normalized Difference Vegetation Index | NDVI_16_day | Dimensionless |  | 16 days  250 meters |
| Gridded population density | pop_density | number of people per square kilometer | Socioeconomic Data and Applications Center, NASA  <https://sedac.ciesin.columbia.edu/data/set/gpw-v4-population-density-rev11> | Spatial: 1Km |
| Land cover | land_cover | --- | CCI Land Cover (LC) team, European Space Agency  <https://2016africalandcover20m.esrin.esa.int/> | Spatial: 20 meters |
| Elevation | elevation | meter | U.S. Geological Survey  <https://maps.princeton.edu/catalog/stanford-jm998sr5227> | Spatial: 450 meters |

Table S2. Summary of mosquito sampling effort in Mozambique and Ghana by surveillance design. HLCs were conducted both indoors and outdoors. CDC LTs were placed indoors while CDC LTs+tent collections were conducted outdoors. Indoor Prokopack collections were used to sample resting mosquitoes.

| **Country** | **Province / District** | **Year** | **EASF** | | **Routine** | | **Collection methods** |
| --- | --- | --- | --- | --- | --- | --- | --- |
|  |  |  | **No. of houses** | **Sampling effort** | **No. of houses** | **Sampling effort** |  |
| **Ghana** | Volta | Year 1 | 48 | 288 | 8 | 100 | HLCs |
|  |  |  | 120 | 708 | 20 | 238 | PSCs |
|  | Greater Accra | Year 1 | 40 | 268 | 8 | 112 | HLCs |
|  |  |  | 85 | 556 | 19 | 113 | PSCs |
|  | Western north | Year 1 | 40 | 395 | 8 | 16 | HLCs |
|  |  |  | 100 | 788 | --- | --- | PSCs |
| **Mozambique** | Niassa / Cuamba | Year 2 | 15 | 42 | 6 | 23 | HLCs |
|  | Niassa / Mandimba | Year 2 | 12 | 114 | 6 | 11 | HLCs |
|  | Zambezia / Morrumbala | Year 1 | 12 | 323 | 14 | 128 | CDC LTs and CDC LTs+tent, Prokopack |
|  |  | Year 2 | 12 | 529 | 16 | 136 | HLCs, CDC LTs, Prokopack |

Table S3. Root mean square standardized prediction error (RMSSPE) for predicting the number of collected mosquitoes using data from the EASF and Routine designs.

| **Country** | **Prediction** | **Average** | **95% confidence interval** |
| --- | --- | --- | --- |
| **Ghana** | **EASF data predicting Routine locations** | 6.17 | (5.46, 6.99) |
|  | **Routine data predicting EASF locations** | 5.67 | (4.17, 8.53) |
| **Mozambique** | **EASF data predicting Routine locations** | 1.267 | (0.740, 2.067) |
|  | **Routine data predicting EASF locations** | 4.234 | (1.114, 11.502) |

Table S4. Total number and proportion of obsolete locations by country and surveillance design.

| **Country** | **Design** | **Number (percentage) of obsolete surveillance locations after 5 months** | **Number of collections potentially saved if obsolete location is removed** |
| --- | --- | --- | --- |
| Ghana | EASF | 6 (5%) | 25 |
|  | Routine | 8 (33%) | 16 |
| Mozambique | EASF | 15 (16%) | 108 |
|  | Routine | 6 (30%) | 14 |

Table S5. Root mean square standardized prediction error (RMSSPE) for predicting the number of collected mosquitoes using data from the EASF and Routine designs. *Not calculated because of no repetitions.

| **Country** | **Prediction type** | **Average error** | **95% confidence interval** |
| --- | --- | --- | --- |
| **Ghana** | **Routine data predicting leaved-out Routine locations** | 5.13 | NA* |
|  | **EASF data predicting leaved-out EASF locations** | 3.81 | (2.76, 5.51) |
| **Mozambique** | **Routine data predicting leaved-out Routine locations** | 0.831 | NA* |
|  | **EASF data predicting leaved-out EASF locations** | 1.663 | (0.293, 4.741) |

Table S6. EASF model evaluation between year 1 and year 2 for EASF in Mozambique and between 6 and 12 months in Ghana. PMCC: Predictive Model Choice Criteria, CV coefficient of variation. Modified signed-likelihood ratio test (M-SLRT) has been applied to test the difference between CVs. *No test is applied to the PMCC difference due to the nature of this information criteria.

| **Country** | **Statistic** | **Difference (%)** | **Test** |
| --- | --- | --- | --- |
| **Ghana** | **CV** | -47 | <0.001 |
|  | **PMCC** | +101 | NA* |
| **Mozambique** | **CV** | -99 | <0.001 |
|  | **PMCC** | -87 | NA* |
